# Adherence to the Mediterranean Diet by Greek Patients With Glaucoma: An Observational Study

**DOI:** 10.7759/cureus.108435

**Published:** 2026-05-07

**Authors:** Ioanna C Vlachogianni, Klio I Chatzistefanou, Konstantinos Droutsas, Marilita M Moschos

**Affiliations:** 1 1st Department of Ophthalmology, National and Kapodistrian University of Athens, Athens, GRC

**Keywords:** adherence, bmi, fat mass, glaucoma, mediterranean diet

## Abstract

Purpose: This study aimed to study adherence to the Mediterranean diet (MedDiet) by patients diagnosed with primary open-angle glaucoma (POAG).

Methods: An observational study was conducted to assess adherence to the MedDiet by patients with POAG. Patients attended the Ophthalmology Department of the General Hospital of Athens “Georgios Gennimatas” in Athens, Greece. The study included completing an 11-item questionnaire validated by Panagiotakos et al. in person.

Results: A total of 355 volunteers successfully completed the questionnaires, 168 patients in the control group and 187 patients with glaucoma. In men with glaucoma, adherence to the MedDiet was low in 14 patients (14%), moderate in 67 (67%), and high in 19 (19%) cases. In women with glaucoma, adherence was low in 17 patients (19.5%), moderate in 52 (60%), and high in 18 (20.7%) cases. The overall adhesion to the MedDiet by patients with glaucoma is low in 16.6%, moderate in 63.6%, and high in 19.8% of the cases. According to the body mass index (BMI) classification, the patients categorized in the higher obesity level have lower adherence to the MedDiet (p = 0.025). Patients with glaucoma who have higher adherence to the MedDiet have lower weight, waist circumference, and fat mass (p < 0.05).

Conclusion: Greek patients with POAG have moderate adherence to the MedDiet. Future research should focus on MedDiet intervention studies on glaucoma patients in order to assess its effects on modulating disease activities and processes.

## Introduction

The Mediterranean diet (MedDiet) is rooted in the cultural traditions of the countries around the Mediterranean basin [1]. The MedDiet, first described by Ancel Keys in the 1960s, is based on the eating habits of Greeks and Italians. The MedDiet is characterized by a high intake of plant foods (like fruits, vegetables, legumes, nuts, and seeds); minimally processed, seasonally fresh foods; daily intake of extra-virgin olive oil; a moderate intake of dairy products, fish, and poultry; and a low amount of red meat. Moreover, moderate alcohol consumption, mainly wine, is a basic component of this diet [2]. Greater adherence to the MedDiet has many health benefits in chronic diseases, such as type II diabetes, cardiovascular diseases, cancer, and cognitive-related diseases, and overall mortality, as described in the literature [3].

Glaucoma is a leading cause of irreversible blindness worldwide; almost half of glaucoma cases are undiagnosed, and the prevalence is increasing over time [4,5]. The most common type of glaucoma diagnosis is primary open-angle glaucoma (POAG) [6]. The research community knows that there are some risk factors, such as age (above 60 years old), family history, steroid usage, diabetes, high myopia, hypertension, central cornea thickness, and eye injury. Epidemiological data predict that patients with glaucoma will reach 111.8 million worldwide by 2040 [7].

Glaucoma is a chronic neurodegenerative disease with a genetic background, but these days, environmental factors, such as diet, also seem to play an important role in the disease’s progress. The MedDiet could prevent age-related eye diseases, like cataract and glaucoma [8]. According to Rotterdam's study, greater adherence to the MedDiet was significantly associated with a lower incidence of open-angle glaucoma (OAG) [9]. Moreover, high adherence to the MedDiet and lifestyle is a protective factor for glaucoma incidence [10]. Some studies have reported the protective effect of a healthy diet on glaucoma progression [11], which includes green leafy vegetables, omega fatty acids, and moderate intake of hot tea and coffee, or a diet rich in antioxidants, such as the MedDiet [12].

Relying on these data, we performed a study on patients with POAG in order to estimate their adherence to the MedDiet. To the best of our knowledge, there are no data about the adherence of Greek patients with glaucoma to the MedDiet.

## Materials and methods

Study design

The observational research took place from May 2018 to December 2021 at the Ophthalmology Department of the General Hospital of Athens “Georgios Gennimatas” in Athens, Greece. The study included patients diagnosed with POAG and excluded patients with other ocular diseases. The study recruited 400 volunteers, 200 volunteers for the control group (age-matched) and 200 patients for the glaucoma group. For the control group, volunteers were age-matched healthy individuals without eye diseases. However, some of them did not meet the inclusion criteria or did not complete the study. Finally, we included 168 volunteers in the control group and 187 patients in the glaucoma group. The major limitation of this study is that it is an observational case-control study at a single time point.

Data were obtained through structured standard questionnaires administered by trained personnel (see Appendix). Information on lifestyle (including physical activity, tobacco smoking, and dietary supplement usage) was collected. The questionnaires and anthropometric measurements were administered by a single dietitian in a calm and relaxed environment and lasted 30 minutes. In any case, patients who did not meet the glaucoma criteria, who had memory problems, and who failed to complete all the required questionnaires were excluded from the study. The study protocol was approved by the Scientific Board of the General Hospital of Athens “Georgios Gennimatas” (approval no. 25219/8-2018), and it was carried out in accordance with the Declaration of Helsinki [13].

Anthropometric measurements

Measurement of Weight and Fat Mass

All weight measurements were done with the same Tanita MC 580 machine (multi-frequency body composition analyzer with BIA; Tanita, Tokyo, Japan). Weight/fat mass measurements were done two times; if there was a difference of more than 100 g in the weighing or 1% in the fat measurement, the measurement was repeated a third time. Measurements of weight and fat mass were conducted only if the subjects followed the protocol: 12-hour fasting and avoiding caffeine for four hours, alcohol, and exercise for 24 hours. All the volunteers could drink a normal amount of water in the 24 hours prior but avoid excessive hydration or dehydration. A high percentage of fat mass was defined as values >25% for men and >35% for women, as suggested by the World Health Organization (WHO) [14].

Height Measurement

Height measurement was performed with a simple Seca 206 stanchion mounted on the wall (Seca, Hamburg, Germany). The measurement was done two times; if there was a difference of more than 1 cm, the measurement was repeated a third time.

Determination of the Body Mass Index (BMI)

The BMI is calculated as the ratio of weight in kilograms to height in meters squared: BMI = weight (kg) / (height)^2^ (m^2^). The volunteers were categorized by BMI according to the classification criteria of the WHO. The volunteers were categorized as follows: BMI = 18.5-24.9 kg/m^2^ (normal); 25-29.9 kg/m^2^ (overweight); 30-35 kg/m^2^ (obesity I); 35-40 kg/m^2^ (obesity II); and >40 kg/m^2^ (obesity III) [15].

Waist, Abdominal, and Hip Circumference Measurement

The waist measurement is defined as the point midway between the iliac crest and the lower rib. The abdominal measurement is taken at the height of the iliac crest, typically level with the umbilicus. The hip measurement is the imaginary line that surrounds the buttocks and pelvis. A non-elastic tape calibrated in mm (Seca 201) was used for the measurement. The measurements were made in the same conditions as the weight. High waist circumference values were identified as values >102 cm in men and >88 cm in women [16]. Central obesity is categorized in men with a waist circumference >94 cm and women >80 cm. Abdominal obesity is categorized in men with a WHR >1 and women >0.8.

Questionnaire to assess the level of adherence to the Mediterranean diet

We used the MedDiet Score, the questionnaire validated for the Greek population by Panagiotakos et al. (Appendix A) [17]. This scale provides a more detailed assessment, assigning scores from 0 to 5 for each item due to the frequency of consumption, allowing for higher granularity in measuring traditional food consumption, including non-refined cereals, potatoes, and alcohol. By summing up, a score is obtained; the higher the score, the greater the adherence to the MedDiet pattern. Volunteers with a score of up to 25 had low adherence to the MedDiet, up to 35 moderate, and up to 55 high.

Athens Physical Activity Questionnaire (APAQ) to assess physical activity

This questionnaire has also been validated for the Greek population (Appendix B) [18]. Physical activity is divided into three categories: physical activity at work, at home, and in social life. Accurate information is given on the hours and type of exercise, to check if there is a sedentary lifestyle. Finally, physical activity was categorized based on metabolic equivalents (METs) into three categories, i.e., PAL = 1.3, 1.5, and 1.7, and the hours of exercise based on the questionnaire. Calories per minute of exercise were calculated using the formula: (MET x 3.5 x weight in kilograms)/200.

Statistical analysis

Statistical analysis was performed using the statistical package IBM SPSS Statistics for Windows, version 28.0.1.0 (IBM Corp., Armonk, NY). The sample size was calculated based on the primary data of BMI values. We set the single power value of 0.9; population mean difference of 1, which is equal for the two groups; and standard deviation of 3. The result was 191 subjects per group. Results were expressed as mean ± standard deviation (SD) or median and min-max range, as stated. Differences between groups for scale variables were estimated using the unpaired Student’s t-test. The chi-square test was used to detect differences between nominal-ordinal parameters. A p-value <0.05 was considered statistically significant.

## Results

Basic anthropometric characteristics of the study population

Anthropometric Measurements and Body Composition

The control group consists of volunteers with age of 71.62 ± 9.5 years, weight of 78.4 ± 14.79 kg, height of 1.65 ± 0.098 meters, % body fat of 33.58 ± 8.12, fat mass of 58 ± 8.97 kg, % total body water of 46.05 ± 5.27, and BMI of 28.64 ± 6.34 (Table 1).

**Table 1:** Descriptive characteristics of the participants.

Group		Min	Max	Mean	Std. dev.
Control	Age (years)	41	92	71.62	9.50
	Weight (kg)	46.3	150.0	78.42	14.79
	Height (m)	1.47	1.90	1.65	.10
	% body fat	19	51	33.49	7.99
	% total body water	35	56	46.12	5.24
	Waist (cm)	59	137	96.99	11.63
	Abdominal (cm)	80	140	104.90	10.54
	Hip (cm)	82	132	104.90	9.68
	ΒΜΙ (kg/m^2^)	18.09	46.30	28.64	4.40
	WHR	.62	1.06	.93	.08
	Fat mass (kg)	11.23	55.50	26.58	8.97
Glaucoma	Age (years)	23	91	70.78	10.32
	Weight (kg)	47.0	154.0	76.32	15.83
	Height (m)	1.45	1.90	1.65	.10
	% body fat	15	50	31.89	7.67
	% total body water	35	59	47.15	5.05
	Waist (cm)	60	129	95.38	12.71
	Abdominal (cm)	71	144	103.41	11.78
	Hip (cm)	76	140	103.78	10.69
	ΒΜΙ (kg/m^2^)	18.95	48.61	27.94	4.76
	WHR	.68	1.08	.92	.08
	Fat mass (kg)	7.05	51.55	24.43	8.46

In the percentage analysis, 22% of the sample were normal in terms of BMI classification, 46.4% were overweight, and 31.5% were obese. The distribution among women and men is 51.8% and 48.2%, respectively. Regarding the anthropometric waist circumference, the average value was 97.04 ± 11.69 cm, the abdominal circumference was 104.95 ± 10.55 cm, the hip circumference was 106.22 ± 10.06 cm, the waist/hip ratio (WHR) was 0.87 ± 0.082, and the waist circumference/height (WHtR) was 0.59 ± 0.068. Specifically, with regard to the WHO, our data show that only 18.45% had a waist circumference measurement within normal limits.

The group of glaucoma patients has the following characteristics: 70.78 ± 10.31 years, weight 76.31 ± 15.83 kg, height 1.65 ± 0.10 meters, % body fat 31.87 ± 7.9, fat mass 24.43 ± 8.46 kg, % total body water 47.16 ± 5.16, and BMI 28.64 ± 6.34. In the percentage analysis, 30% of the patients are classified as normal, 39.6% overweight, and 30.5% obese. The distribution among women and men is 46.5% and 53.5%, respectively. Waist circumference averaged 95.56 ± 12.74 cm, abdominal circumference 103.80 ± 11.66 cm, hip circumference 106.35 ± 10.94 cm, waist/hip circumference ratio (WHR) 0.86 ± 0.077, and waist circumference/height (WHtR) 0.58 ± 0.077 (Table 1). Moreover, 27.27% of the patients with POAG are within normal limits in terms of the waist circumference.

**Figure 1:**
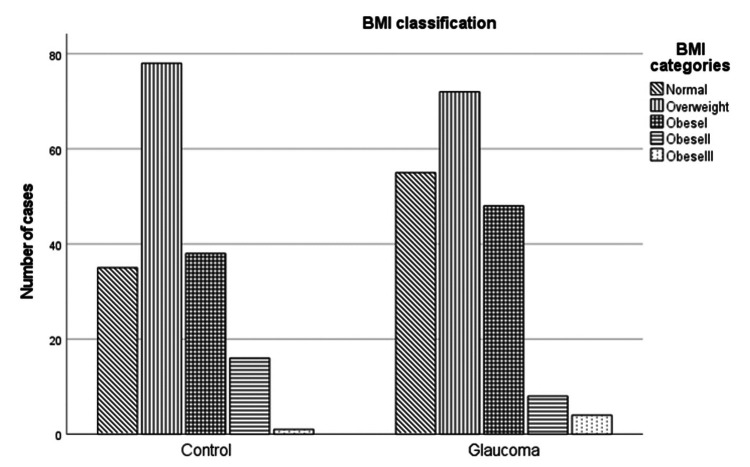
Body mass index (BMI) categorization in both groups.

Regarding anthropometric measurements, the only parameter that differentiates is the fat mass, with statistical significance between the two groups; patients in the glaucoma group have less fat in their bodies (p = 0.017). All other variables did not differ between the two groups. The distribution of the groups by BMI classification (normal, overweight, obese I, obese II, and obese III) is presented in Figure 1.

In the control group, the percentage of people categorized in the obese I category was 22.6%, obese II 9.5%, and obese III 0.6%. The rates in the group of patients with glaucoma are similar, i.e., 25.7% (obese I), 4.3% (obese II), and 2.1% (obese III). There is a difference between groups in the normal and obese II categories. In the control group, there are fewer people in the normal category and more in obese II (p = 0.038). When we add sex as a confounding factor, we show that women in the normal BMI category are more in the glaucoma group (p = 0.04) and men in the obese II category are more in the control group (p = 0.043).

Another categorization for obesity is by fat mass: 75% and 53% women in the control group and glaucoma group are obese, respectively. In both groups, women who are considered obese in terms of fat mass are statistically more than non-obese (p = 0.022). Moreover, 67% of men in the control group and 61% in the glaucoma group are obese by fat mass; there is no statistical difference between the two groups. For women, 85% of the control group and 75% of the glaucoma group have central obesity, and for men, the respective percentages are 76% and 65%, but there is no statistical difference between the groups. Based on the abdominal obesity criteria, there is no statistically significant difference between the two groups for men and women. 

Adherence to the Mediterranean diet

**Figure 2:**
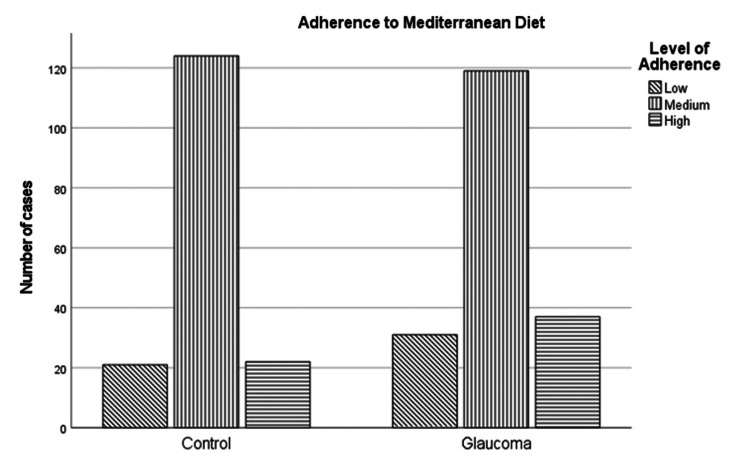
Adherence to the Mediterranean diet (MedDiet) between the two groups, calculated by the MedDiet score.

The MedDiet was assessed in this study with the MedDiet score, which is an easy and quick questionnaire. The score was used to assess the level of adherence to the MedDiet pattern. The results are presented in Figure 2.

In the control group, the mean score was 30.69 ± 4.64, ranging from 19 to 44, with Q1 at 28, Q2 at 31, and Q3 at 34. In the control group, adherence to the MedDiet was low in 12.5%, moderate in 74.4%, and high in 13.1%.

In the glaucoma group, the mean score was 30.57 ± 5.24, ranging from 15 to 45, with Q1 at 27, Q2 at 30, and Q3 at 34. Adherence to the MedDiet pattern was low in 16.6%, moderate in 63.6%, and high in 19.8%.

The MedDiet score mean value does not differ between the two groups, but neither does the level of adherence.

If we split groups by sex, adherence to the MedDiet in males with glaucoma was low in 14 patients (14%), moderate in 67 (67%), and high in 19 (19%) cases. In women with glaucoma, adherence was low in 17 patients (19.5%), moderate in 52 (60%), and high in 18 (20.7%) cases. Adherence to the MedDiet in males in the control group was low in nine patients (11%), moderate in 57 (70.4%), and high in 15 (18.5%). The respective percentages for women are low in 12 patients (13.8%), moderate in 68 (78.2%), and high in seven (8%). There is a statistically significant difference for women in the glaucoma group; more women had high adherence to the MedDiet than the control group (p = 0.042).

Relationship Between Levels of Adherence to the MedDiet With Anthropometric Measurements 

In Table 2, the basic anthropometric data are expressed by the group and level of adherence to the MedDiet. Glaucoma patients with moderate adherence to the MedDiet have less fat mass (p = 0.028) and more total water in their bodies in comparison with the control group (p = 0.031). Moreover, the patients with high adherence have less body weight and abdominal circumference. There is no difference between the BMI criteria and MedDiet adherence, but the higher the BMI, the fewer people who have high adherence to MedDiet, in both groups. 

**Table 2:** Anthropometric parameters between the two study groups separated by the level of adherence to MedDiet. AdMedDiet = adherence to the Mediterranean diet. *p < 0.05.

	Low AdMedDiet		Moderate AdMedDiet		High AdMedDiet	
	Control	Glaucoma	Control	Glaucoma	Control	Glaucoma
Age (years)	73.5 ± 10.1	69.5 ± 12.1	71.7± 9.6	70.7 ± 10.5	69.3± 8.1	72 ± 7.8
Weight (kg)	83.2 ± 19.5	77.4 ± 14.8	77.1 ± 14.3	76.8± 14.8	81.3 ± 10.9	73.7± 12*
Height (m)	1.64 ± 0.12	1.63 ± 0.1	1.65 ± 0.1	1.65 ± 0.1	1.70 ± 0.07	1.65 ± 0.1
% Fat	36.2 ± 7.4	33.7 ± 8.7	33.6 ± 8.4	31.5 ± 7.8*	30.9 ± 6.8	31.7 ± 7.7
% Water	44.6 ± 4.2	46.4 ± 5.9	45.6 ± 6.9	51.4± 7.8*	47.7± 4.3	47.1± 5
Waist (cm)	101.3 ± 13.3	98.6 ± 12.7	95.8 ± 11.7	94.8 ± 13.6	98.8 ± 9.2	95.4 ± 9.9
Abdominal (cm)	109.6 ± 12	107.8 ± 10.8	103.8± 10.5	103 ± 12.5	106.7± 7.8	102.5 ± 9*
Hip (cm)	109 ± 9.9	111 ± 12.4	105.5 ± 10.3	105.4± 11.2	108.8 ± 6.4	104.7± 7.9
BMI (kg/m^2^)	30.76 ± 5.5	29.1 ± 4.9	28.4 ± 4.3	28± 5	28.2 ± 3.4	26.9± 3.7

Relationship between levels of adherence to the MedDiet and physical activity

Concerning the physical activity of the control group, 67.3% were characterized with low physical activity, 25.6% with moderate, and 7% with high physical activity. For the assessment of physical activity, the METs of the type of exercise and its duration were evaluated. In the glaucoma group, 61.5% of the patients have low physical activity, 30% have moderate physical activity, and 8.6% have high-intensity and high-duration physical activity. There is no difference between MedDiet adherence and physical activity level. If we analyzed the types of exercise, there is a statistical difference between the two groups for those with moderate adherence to the MedDiet (Table 3). These people spend more hours per week on light (p = 0.023) and heavy housework (p = 0.025) and less on reading (p = 0.021).

**Table 3:** Sleep and physical activity parameters between the two study groups separated by the level of adherence to the MedDiet. AdMedDiet = adherence to the MedDiet. *p < 0.05.

	Low AdMedDiet		Moderate AdMedDiet		High AdMedDiet	
	Control	Glaucoma	Control	Glaucoma	Control	Glaucoma
Sleep hours_perday	6.2 ± 1.9	7.8±13	6.5 ± 1.9	6.4 ±2	7.1 ± 1.4	6.6 ± 1.5
WatchTv hours_perday	3.2 ± 2.3	5±6.7	3.6 ± 5.1	3.3 ± 4.2	3.8 ± 4.9	3.4 ± 2.9
Lighthousework hours_per week	6.2 ± 8.1	10.3±11.9*	6.2 ± 8.8	9 ± 10.6*	7.4 ± 9.6	8.8 ± 9.9
Heavyhousework hours_perweek	0.4 ± 0.82	1.4±3.7*	2.4 ± 5	4 ± 2*	3.9 ± 8	2.3 ± 4.5
Reading hours_perweek	7.1 ± 8.6	6.7 ± 9.6	7.3 ± 11.3	4.6 ± 7.2*	5.6 ± 9.1	6.3 ± 7.4
Working hours_perweek	12.7 ± 24.2	11.1 ± 22.4	9.4 ± 23.8	5.8 ± 17*	12.4 ± 24.4	1.5 ± 9.2*
Workingout hours_perweek	1.5 ± 3.4	1.8 ± 2.4	2.3 ± 3.5	2.9 ± 4.2	3.4 ± 2.8	2.8±3.7

Lifestyle habits

In terms of smoking habits, in the control group, 20.8% were current smokers, 39.3% were ex-smokers, and 39.9% were never smokers. In the glaucoma group, 18.2% were current smokers, 37% were ex-smokers, and 45.45% were never smokers. It is also important to note that 30.4% of the sample consume nutritional supplements. According to the data, the most frequently consumed dietary supplements are, in order of preference, vitamin D, omega-3 fatty acids, magnesium, aloe vera gel, and B vitamins. In the glaucoma group, 40.9% consume dietary supplements, which means that glaucoma patients who consume dietary supplements are more than the control group (p = 0.01). According to the data, the most frequently found nutritional supplements are, in order of preference, vitamin D, omega-3 fatty acids, magnesium, aloe vera gel, and B vitamins. Dietary supplement consumption does not affect dietary habits because there is no difference in the level of adherence to the MedDiet.

## Discussion

Body composition

Obesity is a chronic disease and a major public health problem; it has a growing worldwide prevalence, irrespective of age, sex, race, or socioeconomic status [19]. Obesity is a health problem that affects the whole body and the ophthalmic system. The WHO defines obesity as abnormal or excessive fat accumulation that poses an increased risk to health. There are numerous investigations reporting a significant relationship between obesity and glaucoma. A possible explanation for the mechanism is the high level of oxidative stress at the trabecular meshwork, which blocks the aqueous humor outflow channel and increases intraocular pressure (IOP) [20]. Another suggestion is that obesity causes an intestinal dysbiosis, which could induce systemic inflammation that activates TLR4 and may alter retinal and ocular barriers. The data are still limited, but between BMI and waist-hip circumference, there is a link very useful for glaucoma prevention and treatment [21].

The two study groups differ in terms of the BMI criteria; the control group has more overweight and obese people than the glaucoma group. In detail, more men from the control group were categorized in the obese II category than the control group (p < 0.05), and patients with central obesity were more in the control group, but there was no statistical difference. Therapy lines for glaucoma must also focus their practices to change this because obesity is associated with OAG prevalence [22]. The results of this study showed that patients with glaucoma have less fat mass than controls (Table 4). Moreover, in the glaucoma group, there are more people categorized as normal according to the BMI (Table 4). Previous research showed that there is an association between high fat mass and OAG prevalence [23]. Moreover, excessive fat mass and obesity affect oxidative stress, systemic inflammation, and, finally, retinal-ocular barriers. Jung et al. reported that patients who are metabolically healthy and obese are at higher risk of developing POAG compared to non-obese patients, suggesting that obesity carries some risk for POAG [24].

**Table 4:** Categorization of the main results

	Control	Glaucoma	p value
Body composition			
Women, normal (BMI)	20.7%	32.2%	0.04
Men, obese II (BMI)	6.2%	1%	0.043
Fat mass	26.2 ± 8.97 kg	24.43 ± 8.46 kg	0.017
Mediterranean diet adherence			
Women with high AdMedDiet	8%	20.6%	0.042
Moderate AdMedDiet %FAT	33.6 ± 8.4	31.5 ± 7.8*	0.028
Moderate AdMedDiet %Water	45.6 ± 6.9	51.4 ± 7.8*	0.031
Moderate AdMedDiet Heavyhousework hours_perweek	9 ± 10.6	6.2 ± 8.8*	0.023
Moderate AdMedDiet Reading hours_perweek	7.3 ± 11.3	4.6 ± 7.2*	0.021
Lifestyle habits			
Supplement consumers	30.4%	40.9%	0.01

Mediterranean diet adherence 

This study estimated the level of adherence by glaucoma patients to the MedDiet for the first time. Most patients with glaucoma have moderate adherence to the MedDiet, like the control group, but there is no difference between the two groups. Women with glaucoma have higher adherence than controls (Table 4). That result is in agreement with the fact that women with normal BMI are more in the glaucoma group. The MedDiet improves anthropometric parameters, such as weight and waist circumference [25], and the BMI. When we split groups with adherence, we recognized that there is a difference between adherence anthropometric measurements and physical activity habits. Glaucoma patients with moderate adherence have lower fat mass and move more hours (Table 4), and those with high adherence have lower body weight and lower abdominal circumference. Adherence to the MedDiet could reduce abdominal adiposity and body weight and can be recommended as a healthy diet choice to individuals with a chronic health problem [25].

Lifestyle habits

Smoking habits do not differ between the two groups, but the negative effects of smoking on IOP are known [26]. Smoking is a modifiable risk factor, so doctors and healthcare providers must take into consideration this fact and adopt policies to help glaucoma patients quit smoking. Another outcome from this study is that consumers of dietary supplements are more likely to be in the glaucoma group (Table 4). A possible explanation for this finding is that patients do not follow good nutritional habits and try to enrich their diet via dietary supplements. According to our recent review, patients with glaucoma take supplements rich in vitamins D, E, C, B complex, and omega-3 fatty acids [27]. This observational study confirmed that patients with glaucoma consume supplements mainly with vitamin D, omega-3 fatty acids, and magnesium. Vitamin D supplementation is known for long-term preventive effects on multiple chronic diseases and thus has the potential to decrease all-cause mortality [28]. Moreover, much research concerns about the low blood concentration of vitamin D in glaucoma patients and proposes the consumption of supplements and foods rich in vitamin D. Patients with glaucoma consume supplements with omega-3 fatty acids, because omega-3 fatty acids influence optic nerve blood velocity and modulate systemic microcirculation. Moreover, magnesium is a mineral that improves blood flow. The data of our study support the idea that supplementation of the diet with these ingredients improves eye microcirculation.

## Conclusions

According to the results of this study, we conclude that patients with POAG have moderate adherence to the MedDiet. Women in the glaucoma group have higher adherence to the MedDiet than controls. It is also evident from the results of this study that women with glaucoma maintain normal BMI and fat mass and are more active. In the future, it would be beneficial to include nutritional monitoring and consultation in a holistic approach to the routine clinical management of glaucoma patients, in order to optimize their adherence to the MedDiet. Apart from that, a high MedDiet adherence will affect their overall food-related quality of life and clinical profile.

## Figures and Tables

**Figure 3:**
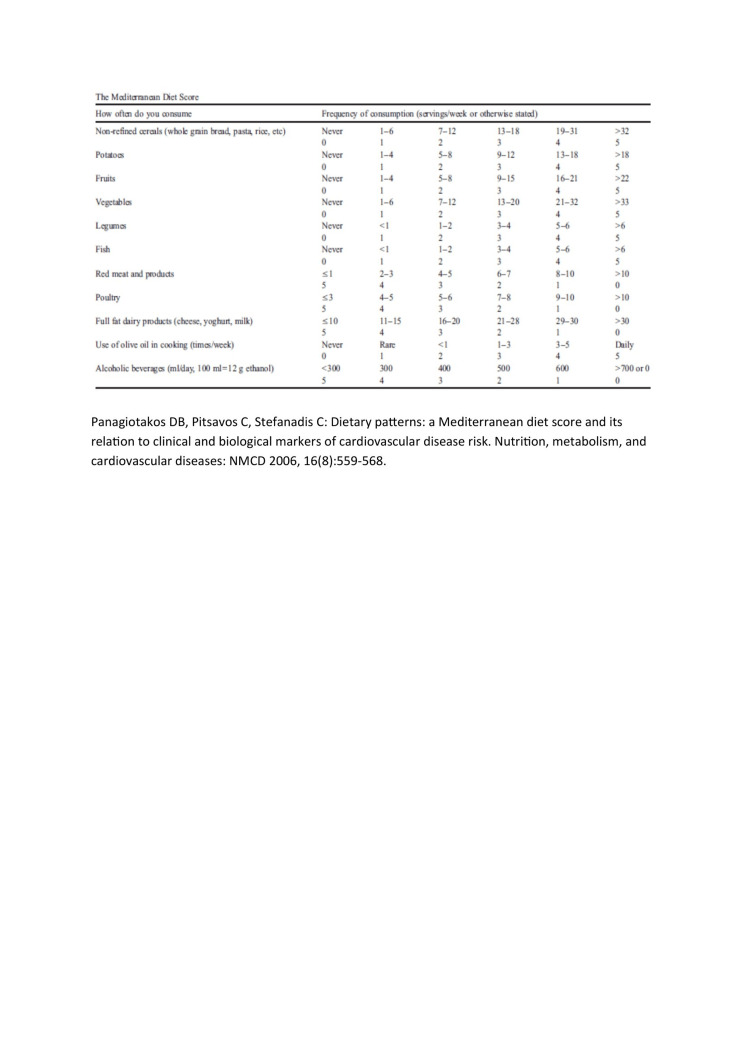
MedDiet Score questionnaire

**Figure 4:**
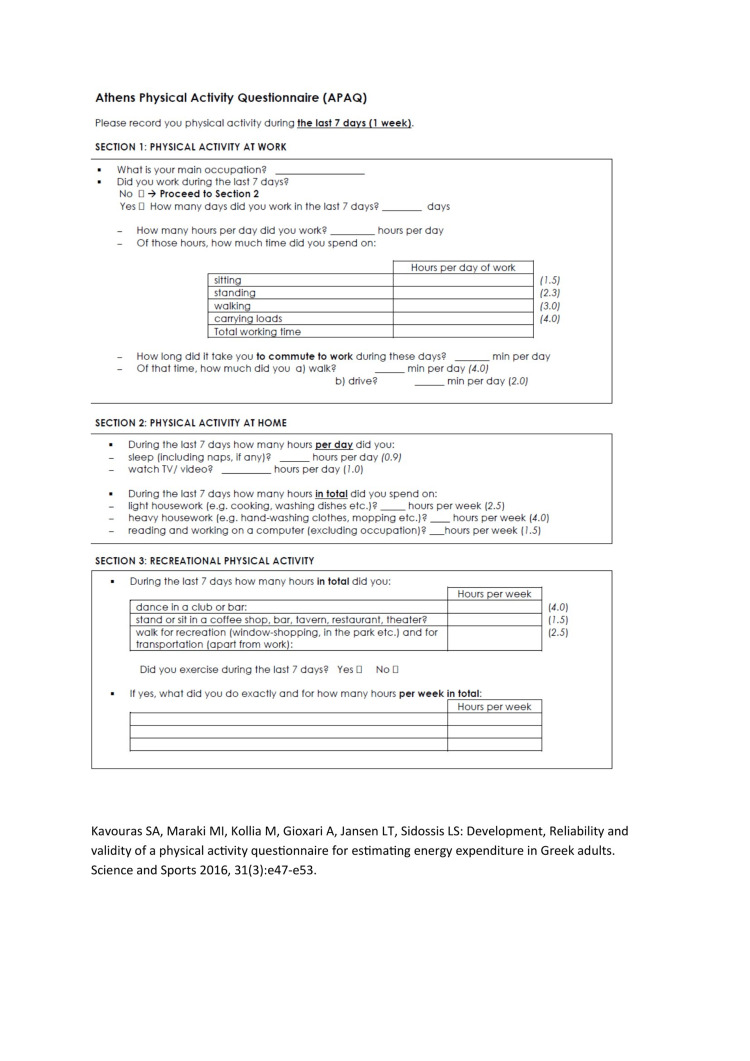
Athens Physical Activity Questionnaire (APAQ)

## Appendices

Appendix A

Appendix B
